# Highly sensitive live-cell imaging-based cytotoxicity assay enables functional validation of rare epitope-specific CTLs

**DOI:** 10.3389/fimmu.2025.1558620

**Published:** 2025-05-08

**Authors:** Kathrin Wellach, Angelika B. Riemer

**Affiliations:** ^1^ Division of Immunotherapy and Immunoprevention, German Cancer Research Center (DKFZ), Heidelberg, Germany; ^2^ German Center for Infection Research (DZIF), Molecular Vaccine Design, Partner Site Heidelberg, Heidelberg, Germany; ^3^ Faculty of Biosciences, Heidelberg University, Heidelberg, Germany

**Keywords:** T cells, cytotoxicity, epitopes, live-cell imaging, rare CTL populations

## Abstract

Many immunotherapeutic approaches aim to induce epitope-specific T-cell cytotoxicity. However, the identification—and especially the functional validation—of suitable epitopes by *in vitro* cytotoxicity assays can be challenging, particularly when the number of available epitope-specific cytotoxic T cells (CTLs) is limited. Here, we present a highly sensitive image-based cytotoxicity assay that allows the functional analysis of rare epitope-specific T cells. The live-cell imaging-based setup combines transient red labeling of target cells with a green caspase 3/7 probe, allowing reliable measurement of the fraction of apoptotic target cells. Time-course analysis enables the monitoring of subtle differences. This highly flexible assay can be applied to assess the killing of either target cells with endogenous epitope presentation or those artificially loaded with the epitope of interest. Analysis of assay sensitivity demonstrated that cytotoxicity mediated by as few as 0.1% epitope-specific CTLs in a T-cell culture can still be detected. The epitope-specificity of the assay was additionally validated by specific upregulation of PD-1 and LAG-3 on epitope-specific T cells, as well as the epitope-specific induction of interferon-γ release. Finally, the assay was successfully applied to functionally validate human papillomavirus (HPV)16 epitopes, by detecting epitope-specific killing of established patient-derived tumor cell lines by rare T-cell populations expanded from peripheral blood. Overall, this cytotoxicity assay setup provides a straightforward approach to assess the cytotoxic capacity of rare epitope-specific T cells and enables the analysis of T-cell responses against endogenously presented epitopes.

## 1 Introduction

The recognition of cancerous or virus-infected cells by cytotoxic T lymphocytes (CTLs) is a crucial prerequisite for disease clearance. Therefore, epitope-centric immunotherapeutic approaches, such as therapeutic vaccination or adoptive T-cell transfer, depend on the induction of a specific CTL response and, thus, on the identification and validation of immunogenic epitopes. It is generally accepted to assess T-cell responses upon activation based on the release of cytokines (usually interferon-γ), proliferation, or the analysis of surface expression of activation markers. However, unlike these surrogates, *in vitro* cytotoxicity assays directly assess the cytotoxic capacity of immune cells, making these assays important tools for the functional validation of T cells. In the past, chromium ^51^Cr release assays (CRA), depending on ^51^Cr-labeling of target cells, were the most commonly applied technique for cytotoxicity assessment (1). Unfortunately, this method has the serious disadvantage of involving radioactive material. Due to this, several other strategies to assess effector cell-mediated cytotoxicity *in vitro* have been developed. These include assays based on alternative, non-radioactive labeling substances (e.g., rare-earth elements) (2, 3), assays measuring the release of intracellular enzymes by dying cells (e.g., lactase dehydrogenase, LDH) (4), and assays based on the uptake of vital dyes by dying cells (5). More recent approaches include flow cytometry-based assays (6, 7), impedance-based measurements (8, 9), and live-cell imaging-based cytotoxicity analyses (10, 11). A major advantage of the impedance- and live-cell imaging-based strategies is their ability to acquire data over time, which enhances sensitivity and reduces the need for multiple effector-to-target cell ratios.

The most common applications of *in vitro* cytotoxicity assays are experiments assessing the functionality of chimeric antigen receptor (CAR) transgenic cells, transgenic T-cell receptor (trTCR) T cells, clonal CTL lines, or unspecifically activated T cells/PBMCs (12–17). In these applications, the frequency of CTLs specific to a single epitope is either very high or not relevant. However, for approaches aimed at identifying or validating disease-specific epitopes, the number of available CTLs—e.g., from peripheral blood—is usually very low. Often, validating the cytotoxicity of such rare CTL populations requires labor-intensive steps to generate or enrich epitope-specific CTLs, such as TCR cloning or sorting with multimers, which limits the number of epitopes and TCRs that can be analyzed.

Nevertheless, the development of cytotoxicity assay setups that allow time-course measurements has opened up the opportunity to directly analyze cytotoxicity mediated by rare epitope-specific CTLs. Here, we describe a highly sensitive cytotoxicity assay setup based on live-cell imaging, which allows the detection of epitope-specific cytotoxicity mediated by as few as 0.1% epitope-specific CTLs within a heterogeneous pool of CD8^+^ T cells.

## 2 Materials and methods

### 2.1 Peptides

Peptides were synthesized by the Research Group GMP & T Cell Therapy of DKFZ, using Fmoc chemistry on a parallel peptide synthesizer with DIC and HBTU activation and OxymaPure as an additive. The crude peptides were purified to > 95% purity by reversed-phase HPLC and characterized by analytical HPLC-MS. Lyophilized peptides were dissolved in DMSO at 10 mg/mL and stored at − 80°C. Peptide sequences and source proteins are given in **Supplementary Table 1**.

### 2.2 Peripheral blood mononuclear cells

Buffy coat preparations of blood from anonymous healthy female donors above 40 years of age were obtained from DRK Blutspendedienst Mannheim. Peripheral blood mononuclear cell (PBMC) isolation was performed using a standard density gradient procedure with Ficoll-Paque™ PLUS (17-1440-02, Sigma Aldrich, Taufkirchen, Germany) in Leucosep tubes (227290, Greiner Bio-One, Kremsmünster, Austria). The isolated PBMCs were washed with 1× PBS, and residual erythrocytes were lysed by incubation with ACK lysis buffer (150 mM NH_4_Cl, 10 mM KHCO_3_, and 0.1 mM EDTA) for 5 min. PBMCs were washed again with PBS, suspended in human serum with 10% DMSO, and cryopreserved in the gas phase of liquid nitrogen. DNA of 5 × 10^6^ PBMCs was isolated with a QIAamp^®^ DNA Mini kit (56304, Qiagen, Hilden, Germany) and sent to DKMS Life Science Lab GmbH (Dresden, Germany) for HLA-typing.

### 2.3 *In vitro* expansion of epitope-specific T cells from PBMCs

For the expansion of epitope-specific T cells, the protocol published by Cimen Bozkus et al. (18) was adopted. In short, PBMCs with known reactivity against CMV, EBV, or HPV epitopes were thawed and washed in 10 mL X-Vivo15 medium (BE02-060F, Lonza, Basel, Switzerland) containing 2 µL Benzonase (E1014-25KU, Sigma). Subsequently, PBMCs were resuspended in X-Vivo15 medium supplemented with 1,000 IU/mL hrGM-CSF (215-GM, R&D Systems), 500 IU/mL IL-4 (204-IL, R&D Systems, Minneapolis, Minnesota, USA), and 50 ng/mL Flt3-L (308-FKN, R&D Systems) and 10^5^ cells/well were seeded in 200 µL in a U-bottom plate (one plate per peptide of interest). The next day, 100 µL medium was exchanged by 100 µL fresh X-vivo15 medium containing 200 ng/mL LPS (tell-3pelps, InvivoGen, Toulouse, France), 20 µM R848 (SML0196, Sigma), 20 ng/mL IL-1β (201-LB, R&D Systems), and 2 µg/mL of the respective peptide of interest. A day later, the cells were fed by exchanging 100 µL medium per well with 100 µL T-cell medium (**Supplementary Table 2**) containing 20 IU/mL IL-2 (200-02, PeproTech, ThermoFisher, Waltham, MA, USA), 20 ng/mL IL-7 (207-IL, R&D systems), and 20 ng/mL IL-15 (200-15, PeproTech). This feeding was repeated every two to three days until day 12. On day 14, two days before the setup of the cytotoxicity assay, the feeding was performed with T-cell medium only. On day 15, the T cells were harvested and CD8^+^ T cells were enriched by untouched magnetic-activated cell sorting (MACS) (130-096-495, Miltenyi, Bergisch Gladbach, Germany). The enriched CD8^+^ T cells were resuspended in T-cell medium overnight and used at day 16 for the cytotoxicity assay.

### 2.4 Flow cytometry analysis of epitope-specific T cells

HLA-A*02:01 u-load Dextramers^®^ (U-LX02 AP 50, Immudex, Virum, Denmark) were prepared according to the manufacturer’s instructions. First, u-load easYmers were loaded with the respective epitope of interest, and the loading efficiency was assessed as described by the supplier (**Supplementary Figure 1**). The easYmers were then coupled to the APC-labeled dextramer backbone. To assess the frequency of epitope-specific T cells after 15 days of *in vitro* expansion, 0.5 × 10^6^ cells were resuspended in 40 µL cell staining buffer (420201, BioLegend, San Diego, California, USA). For activation/exhaustion marker analysis after the cytotoxicity assay, the T cells from four replicate wells per condition were combined and resuspended in 40 µL cell staining buffer. Next, 8 µL of the peptide-loaded dextramers were added to stain for epitope-specific T cells. After 10 min of incubation at RT in the dark, antibodies and the live dead dye were added and incubated for 20 min at RT in the dark. For frequency analysis of epitope-specific T cells, antibodies against CD3 (300448, BioLegend), CD4 (300528, BioLegend), and CD8 (562429, BD, Franklin Lakes, New Jersey, USA) and fixable live/dead NIR dye (L34975, ThermoFisher) were used. For activation/exhaustion marker analysis after the cytotoxicity assay, antibodies against CD8 (563256, BD), PD-1 (130-120-385, Miltenyi), and LAG3 (130-118-677, Miltenyi) and fixable live/dead green dye (L34969, ThermoFisher) were used. After three washes with cell staining buffer, the cells were fixed with 1% PFA for 15 min. Finally, the cells were again washed three times, suspended in 100 µL cell staining buffer, and stored at 4°C in the dark until analysis at a BD FACS Canto II (frequency assessment) or a BD FACS Fortessa flow cytometer (activation/exhaustion marker analysis). Gating strategies are given in **Supplementary Figures 2**, **3**.

### 2.5 Target cell culture

Tumor cell lines were cultured in standard cell culture conditions (37°C, 5% CO_2_) in their corresponding media (**Supplementary Table 2**). CaSki was obtained from ATCC (CRL-1550), and SNU902 and SNU1299 were obtained from the Korean Cell Line Bank. Cell lines were regularly authenticated and confirmed to be free of mycoplasma, SMRV, or interspecies contamination by SNP profiling and multiplex-PCR by Multiplexion GmbH.

### 2.6 Target cell labeling

Cells were harvested, washed twice with pure RPMI medium (21875034, LIFE, ThermoFisher), and resuspended in pure RPMI at 10^6^ cells/mL. Subsequently, 1 µL/mL CellTrackerRed (C34552, ThermoFisher) was added followed by a 15–30-min incubation at 37°C, 5% CO_2_. Incubation times need to be optimized for each cell line. After washing with pure RPMI, cells were resuspended in their respective medium, and culture was continued overnight.

### 2.7 Optimization of target cell detection

To optimize the detection of target cells based on the red fluorescent signal, labeled target cells were harvested one day after labeling and seeded on a flat-bottom 96-well plate in their respective culture medium. The next day, the medium was exchanged for cytotoxicity assay medium (**Supplementary Table 2**), and the plates were monitored in an IncuCyte^®^ SX5 live-cell imaging system (1234, Sartorius, Göttingen, Germany) using a 10× objective in basic mode, acquiring four images per well every 2 h for 48 h. Images were acquired for phase contrast as well as the red fluorescence channel (emission passband: [576, 639]). The IncuCyte2020B software (Sartorius) was used to train target cell detection masks based on the phase contrast images as well as based on the red fluorescence signal (red mask). The phase contrast-based detection was previewed and optimized on three to five pictures of an early and a late time point each, to best detect the area covered by cells. As a starting point for setting the red detection mask, top-hat segmentation with a radius of 100–150 µm, a threshold of 0.5–1 OCU, and edge split-off were used. Based on the performance of these settings, a cleanup with adjustment of the mask size and a hole fill was included. By analyzing the deviation of the red mask from the phase contrast mask, the red masks were refined in an iterative process to achieve optimal detection of each target cell line.

### 2.8 Live-cell imaging-based cytotoxicity assay

Transiently red-labeled target cells were harvested one day after labeling, and 2 × 10^4^ cells per well were seeded on a flat-bottom 96-well plate (167425, Sigma) in their respective culture medium in order to achieve a final density of 40%–70% confluence on the next day. Cultivation at standard cell culture conditions was continued for about 24 h until the cells were properly attached to the plate. On the same day, the epitope-specific *in vitro* T-cell expansion cultures were harvested, and CD8^+^ T cells were enriched by untouched MACS. Enriched CD8^+^ T cells were cultured overnight in T-cell medium.

On the day of the cytotoxicity assay setup, target cells were loaded with peptides when required. For this, the medium of the respective wells on the assay plate was carefully exchanged with cytotoxic assay medium (**Supplementary Table 2**) containing 10 µg/mL of the peptide of interest, while the medium in all other wells was exchanged by assay medium without peptide. The plate was incubated for 2 h at standard cell culture conditions.

To set up the cytotoxicity assay, the enriched CD8^+^ T cells were resuspended at concentrations of 2.67 × 10^5^ cells/mL and 5.3 ×10^5^ cells/mL in assay medium supplemented with 20 IU/mL IL-2, and caspase 3/7 dye (4440, Sartorius) was added with a final dilution of 1:1,200. The medium of the target cells was then removed, and 150 µL of the T-cell suspension was added per well, resulting in 40,000 and 80,000 T cells per well, respectively. Four replicate wells were prepared for each condition. Additionally, one well containing target cells only without caspase 3/7 dye and one well containing T cells only with caspase 3/7 dye were prepared as red and green “single stain” controls for unmixing. The cytotoxicity assays were monitored using an IncuCyte SX5 live-cell imaging system with a 10× objective in basic mode, acquiring four images per well every 2 h for up to 48 h. Images were acquired for phase contrast as well as in the green (emission passband: [494,533]) and red (emission passband: [576, 639]) fluorescence channels.

Image analysis of the obtained data was performed with the IncuCyte2020B software. The target cell detection mask (red mask) was set based on the red fluorescence signal, applying optimized settings for each target cell line, providing the best overlap with phase contrast-based analysis of target cells without T cells. Apoptotic cells were detected based on the green fluorescence channel (green mask) using top-hat segmentation with a radius of 30 µm and a threshold of 2.3 GCU, with the edge split off. If the size of the target cell detection mask was manually increased, the green detection mask was also increased, but by one pixel less. Green and red detection masks were reviewed on three early and three late time point images for each experiment. The overlap mask calculated based on the red and green mask represents the area of apoptotic target cells. Finally, the frequency of apoptotic target cells at each time point was calculated by dividing the area of the overlap mask by the area of the red target cell detection mask:


$$\begin{array}{c} \text{fraction of apoptotic target cells}\\ =\frac{\text{area of green}/\text{red }\left(\text{double positive},\text{ i}.\text{e}.,\text{ apoptotic}\right)\text{ cells}}{\text{area of red cells }\left(\text{all target cells}\right)} \end{array}$$

The calculated frequencies of apoptotic target cells were normalized to *t* = 0 by subtraction. Repeated measures ANOVA was used to test for significant differences between the test condition and the control condition to identify epitope-specific cytotoxicity. Statistical testing was done with R version 4.3.2 using the aov() function. The significance level was set to 0.05.

### 2.9 Multiplexed cytokine detection

Cytotoxicity assay supernatants were analyzed for cytokines using the multiplexed bead-based LEGENDplex™ Human CD8/NK Panel (741187, BioLegend). The assay was performed according to the manufacturer’s instructions. Briefly, 25 µL of cytotoxicity assay supernatant, 25 µL assay buffer, and 25 µL premixed beads were combined per well in a V-bottom 96-well plate and shaken for 2 h at ~ 800 rpm. The beads were washed once with 200 µL wash buffer per well (300×*g* centrifugation), and 25 µL detection antibody was added. After 1 h of incubation with shaking, 25 µL streptavidin-PE was added without washing. The beads were then incubated for another 30 min, washed again with 200 µL wash buffer, and finally resuspended in 150 µL wash buffer. The samples were read on a FACS Canto II on the same day, with 4,200 beads recorded per sample. The acquired data were analyzed using the Data Analysis Software Suite for LEGENDplex™.

## 3 Results

### 3.1 Reliable detection of target cells

**Figure 1:**
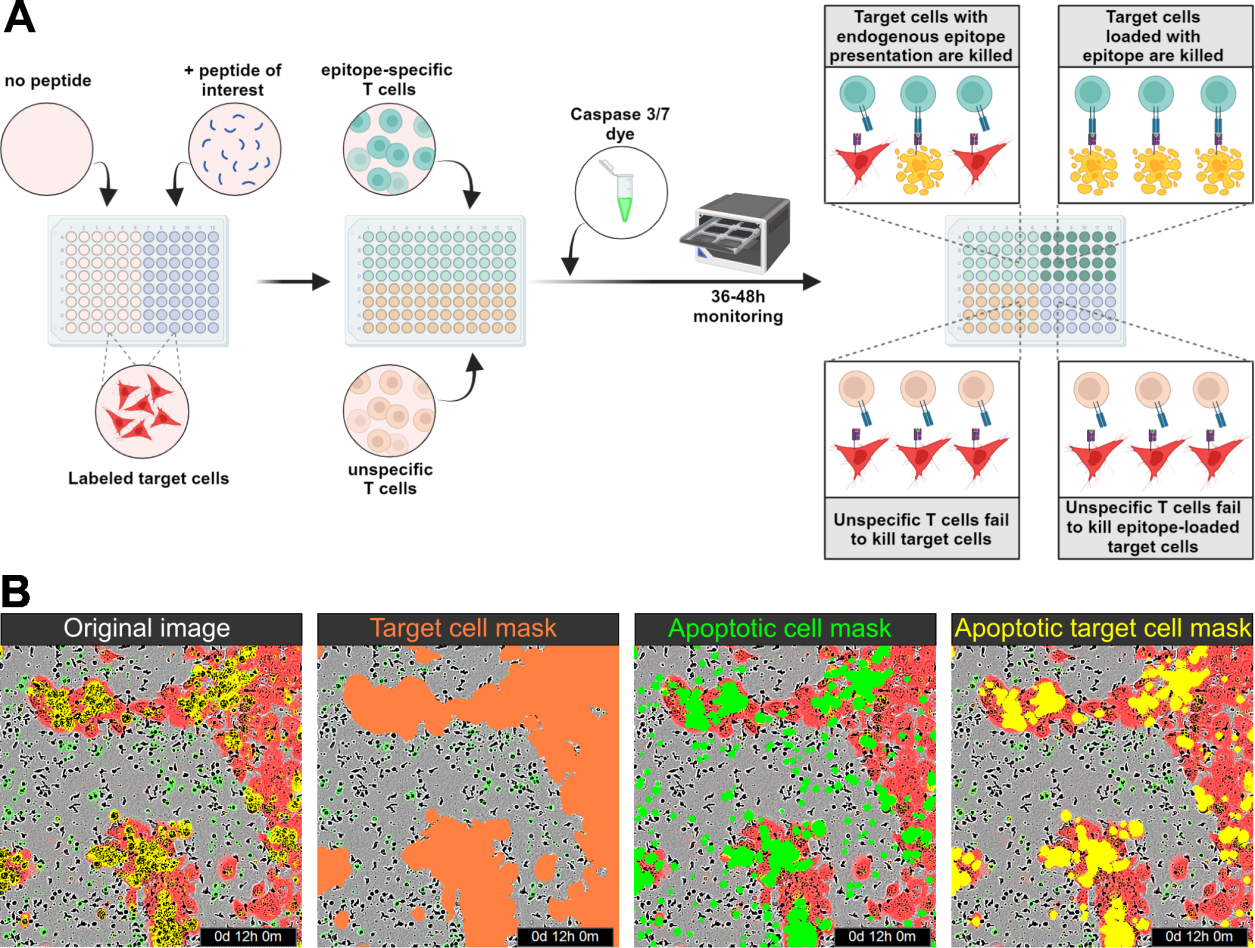
Assay principle. **(A)** Schematic illustration of the assay principle. Transiently labeled target cells are seeded onto the assay plate, and peptide loading is performed if necessary. Epitope-specific and (if required) unspecific T cells, along with a caspase 3/7 dye, are then added to the assay plate, which is monitored for 36–48 h using an IncuCyte system. No epitope-specific killing is observed in co-cultures with unspecific T cells, whereas target cells presenting endogenous epitopes or loaded with peptides are killed by epitope-specific T cells. **(B)** From left to right: Visualization of the original image with red and green fluorescence; red detection mask identifying target cells; green detection mask identifying apoptotic cells; and yellow overlap mask identifying apoptotic target cells.

**Figure 2:**
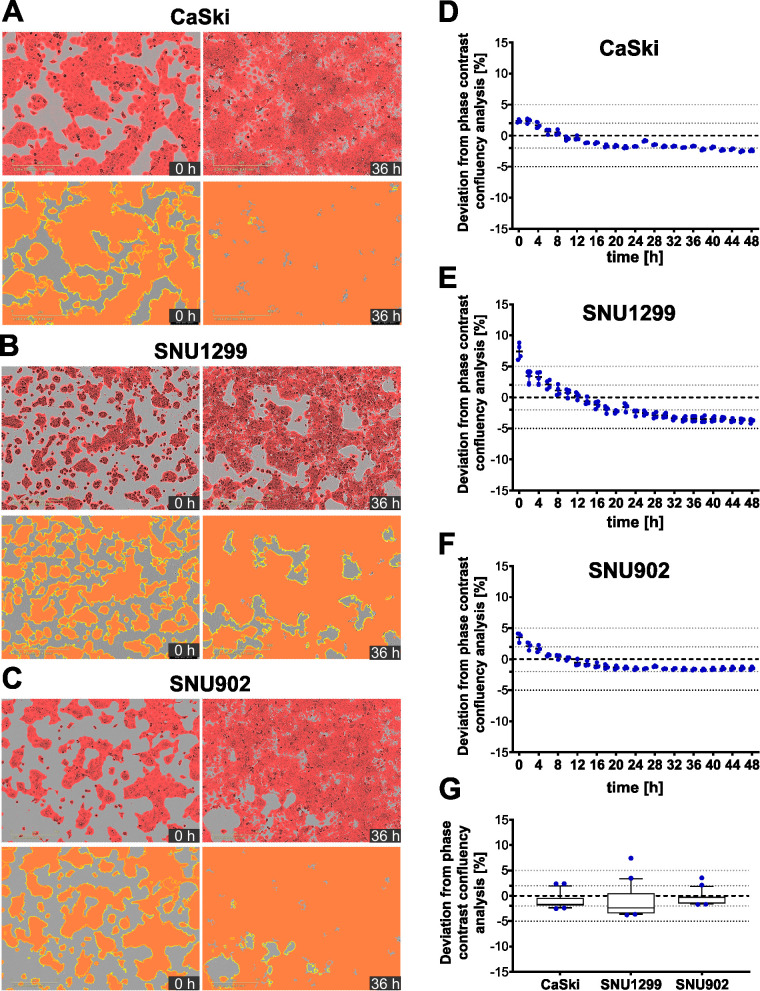
Fluorescence-based detection of target cells compared to phase contrast-based detection. **(A–C)** Representative merged images of phase contrast and red fluorescence channels for transiently red-labeled CaSki **(A)**, SNU1299 **(B)**, and SNU902 **(C)** cells at *t* = 0 h and *t* = 36 h (top), along with corresponding fluorescence-based detection masks (red) and phase contrast-based target cell detection outlines (yellow) (bottom). **(D–F)** Deviation of the red fluorescence mask from the phase contrast-based target cell detection for CaSki **(D)**, SNU1299 **(E)**, and SNU902 **(F)** from *t* = 0 h to *t* = 48 h Four technical replicates and the mean are shown for every time point. **(G)** Summarized data showing the deviation of the red fluorescence mask from the phase contrast-based target cell detection for all three cell lines from *t* = 0 h to *t* = 48 h Whiskers represent the 10th to the 90th percentiles.

This image-based cytotoxicity assay measures T-cell-mediated cytotoxicity by analyzing the fraction of apoptotic target cells upon co-culture with CD8^+^ T cells. For this, transiently labeled target cells are used, which can be loaded with a peptide of interest if applicable. Next, the target cells are co-cultured with CD8^+^ T cells in medium containing a caspase 3/7 dye, which specifically labels apoptotic cells in green. Therefore, apoptotic target cells are red and green double positive, which appears as yellow. Target cell killing is monitored by a live-cell microscope over time (**Figure 1A**). To be able to calculate the fraction of apoptotic target cells, three different image analysis masks needed to be trained to assess the area of target cells (red), apoptotic cells (green), and apoptotic target cells (yellow) (**Figure 1B**). The assay strategy depends on reliable detection of the target cells and differentiation between target cells and T cells, which is enabled by red fluorescence labeling of the target cells. To ensure robust detection of the target cells over time, the target cell area detection based on the phase contrast images as well as based on the red fluorescence signal was compared (**Figure 2**). Here, HPV16^+^ tumor cell lines were chosen as a model system. All cell lines, CaSki, SNU1299, and SNU902, show visually very similar detections for the phase contrast-based analysis (yellow outlines) and the optimized red detection mask (**Figures 2A–C**). The small deviation of the area detected by the red mask from the area detected based on the phase contrast analysis proves good target cell detections over time (**Figures 2D–F**). While the red masks slightly overestimate the target cell area within the first 8–12 h, the fading of the dye over time due to its dilution by target cell division leads to a slight underestimation of the target cell area at later time points. Of note, the deviation of the red mask to the phase contrast analysis relates to cell confluence, leading to higher deviation values at the lower confluence at the beginning of the assay. Summarizing the deviation results of the red masks over the observation period of 48 h shows an average deviation from the phase contrast-based analysis of less than 2% (CaSki, SNU902) or less than 3% (SNU1299), with overall only a slight underestimation of the target cell area (**Figure 2G**). Overall, the red fluorescent labeling is suited to reliably detect the target cell lines over the assay duration.

### 3.2 Detection of epitope-specific T-cell cytotoxicity mediated by common viral epitopes

**Figure 3:**
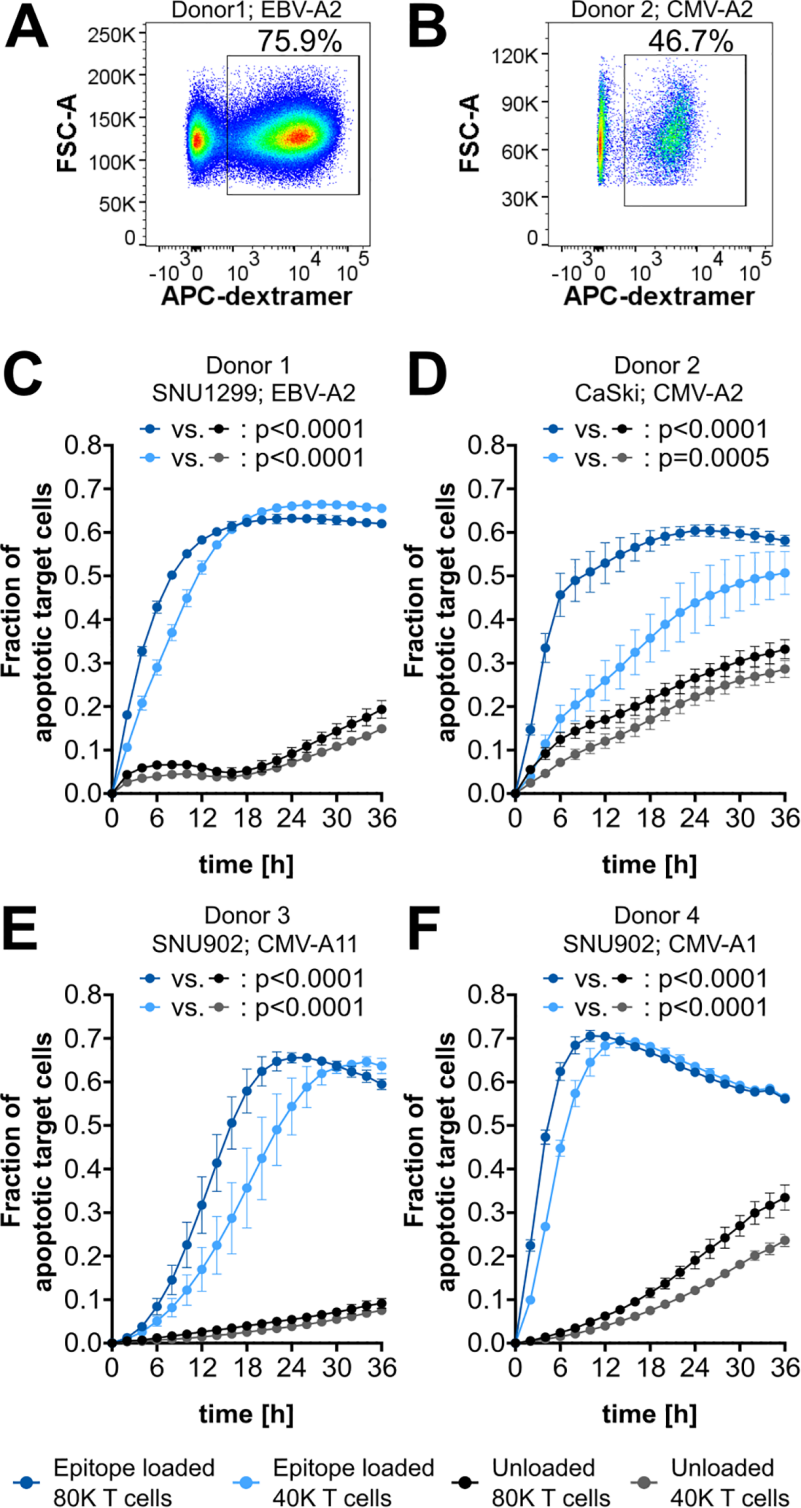
CMV- and EBV-epitope-specific cytotoxicity. **(A, B)** Flow cytometry plots of CD8^+^ T cells with gating for epitope-specific (dextramer^+^) cells. Epitopes and corresponding frequencies are indicated. **(C–F)** Cytotoxicity assay plots showing the fraction of apoptotic target cells after co-culture with 80,000 (80K) or 40,000 (40K) CD8^+^ T cells. Target cells are either loaded with the specific epitope (blue) or left unloaded as a background control (gray). The respective epitopes and target cell lines are indicated. EBV-A2, GLCTLVAM; CMV-A2, NLVPMVATV; CMV-A11, ATVQGQNLK; CMV-A1, YSEHPTFTSQY. The mean of four technical replicates for each timepoint is shown, with error bars indicating the standard deviation (SD). Repeated-measures ANOVA was used for significance testing. *p*-values are shown.

As a first application of the cytotoxicity assay, epitope-specific T-cell cytotoxicity mediated by common epitopes derived from Epstein–Barr virus (EBV) and cytomegalovirus (CMV) was analyzed (**Supplementary Table 1**). *In vitro* expansion of CD8^+^ T cells specific for such epitopes usually yields high frequencies of epitope-specific T cells (19). Also, with our expansion protocol, high frequencies of epitope-specific T cells could be obtained, as shown for the two HLA-A*02:01-binding peptides GLCTLVAML (EBV-A2; 75.9%) and NLVPMVATV (CMV-A2; 46.7%) (**Figures 3A, B**). To assess the cytotoxicity mediated by these *in vitro* expanded T cells, target cells were artificially loaded with the respective peptide, while nonloaded target cells without endogenous peptide presentation were used as a negative control. Upon loading of SNU1299 and CaSki with EBV-A2 and CMV-A2, respectively, strong and highly significant epitope-specific T-cell-mediated cytotoxicity could be observed (**Figures 3C, D**). Similar experiments with the HLA-A*11:01-binding peptide ATVQGQNLK (CMV-A11) and the HLA-A*01:01-binding peptide YSEHPTFTSQY (CMV-A1) also showed strong and specific T cell killing of SNU902 target cells (**Figures 3E, F**). For each of the target cell/epitope combinations, the observed increase in the fraction of apoptotic target cells is delayed in conditions with lower T-cell numbers compared to those with higher T-cell numbers. Notably, the curves may drop at later time points once most target cells are dead or dying. Also, in negative control conditions, the fraction of apoptotic target cells increases over time. This is caused by different factors: a certain amount of apoptotic target cells is normal during the culture of cell lines and might be increased by the transient labeling of the cells. Analysis of the fraction of transiently labeled apoptotic target cells showed that this background level can increase up to 0.1 within 48 h, depending on the target cell line (**Supplementary Figure 4**). Additional background effects show a strong donor and T-cell number dependency, which indicates combined effects of alloreactivity due to the HLA mismatch and the co-culture with high amounts of activated T cells. This is also supported by e*x vivo* experiments with CMV-specific T cells without *in vitro* expansion and activation (**Supplementary Figure 5**), which showed only very low background effects, comparable to the background observed for labeled CaSki without T-cell co-culture. Another aspect that can be observed throughout is that higher T-cell numbers mediate a higher background.

### 3.3 Determination of assay sensitivity

**Figure 4:**
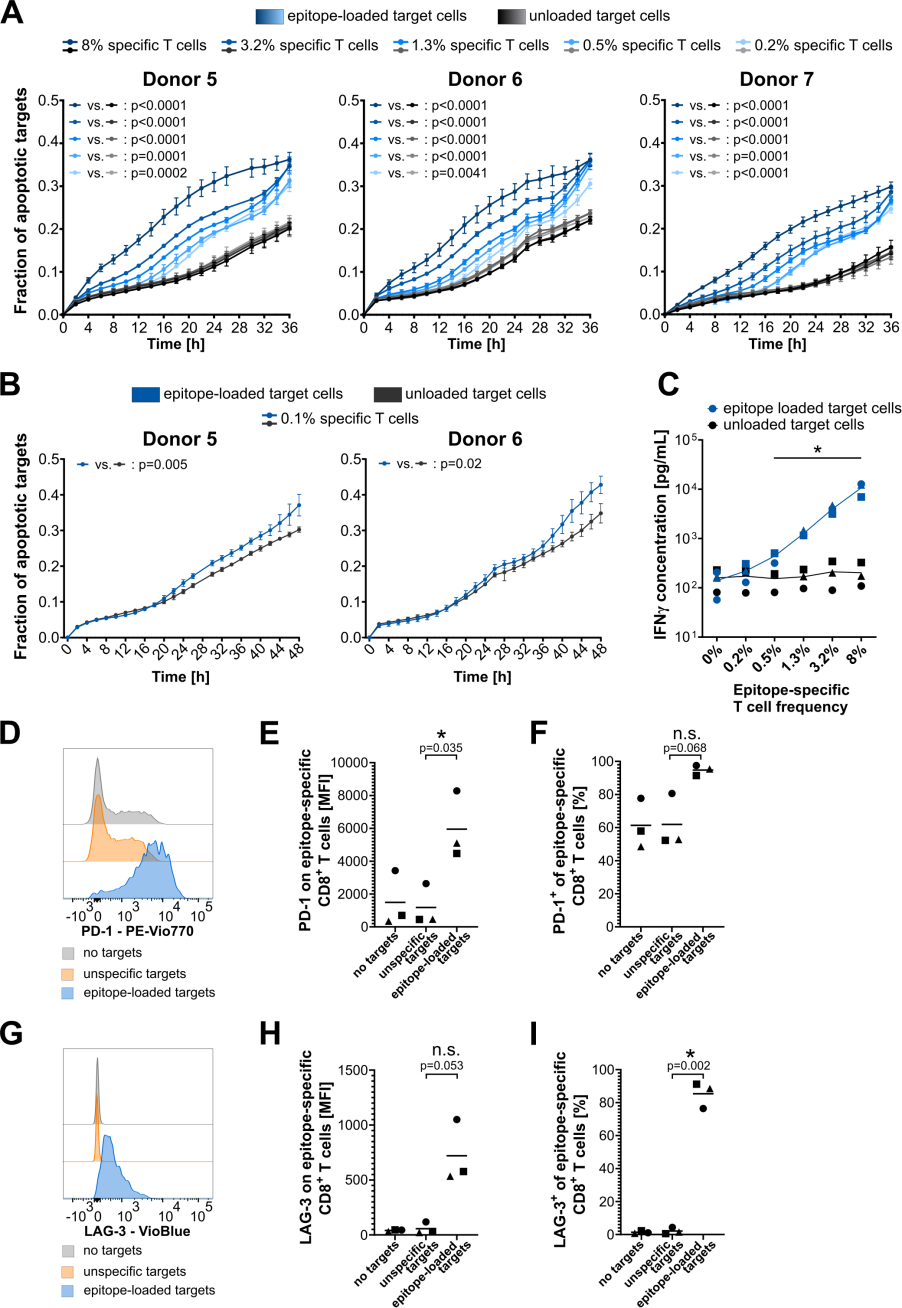
Assay sensitivity and specificity validation. **(A, B)** Cytotoxicity assay plots showing titration of the frequency of epitope-specific CD8^+^ T cells from three **(A)** or two **(B)** independent HLA-A*02:01-expressing donors. Model epitope: EBV-A2 (GLCTLVAML). Target cell line: CaSki. T-cell number per well: 40,000. EBV-A2-specific T-cell frequencies were identified by dextramer staining, and desired frequencies were adjusted by mixing with nonepitope-specific CD8^+^ T cells from the same donor. The mean of four technical replicates for each timepoint is shown, with error bars indicating the SD. Repeated-measures ANOVA was used for significance testing. *p*-values are shown. **(C)** INF-γ concentration in cytotoxicity assay supernatant after 48 h of T-cell–target cell co-culture. Different donors are indicated with different symbols: circles, donor 5; triangles, donor 6; squares, donor 7. Multiple *t*-tests with Holm–Sidak correction for multiple comparisons without assuming consistent SD were used for significance testing in GraphPad Prism 8. Significant differences with *p*-values< 0.05 are highlighted with an asterisk. **(D–F)** PD-1 surface expression on epitope-specific CD8^+^ T cells. **(G–I)** LAG3 surface expression on epitope-specific CD8^+^ T cells. **(D, G)** Exemplary plots for donor 1. **(E, H)** Mean fluorescence intensity (MFI) of PD-1-PEVio770 and LAG3-VioBlue, respectively. **(F, I)** Frequency of PD-1-PEVio770- and LAG3-VioBlue-positive CD8^+^ T cells. **(E, F, H, I)** Different donors are indicated with different symbols: circles, donor 5; triangles, donor 6; squares, donor 7. *t*-tests with Welch’s correction were used for significance testing in GraphPad Prism 8. Significant differences with *p*-values< 0.05 are highlighted with an asterisk; *p*-values are shown. n.s., not significant.

Next, titration experiments were conducted to assess the assay’s sensitivity (**Figure 4**). Effector cell pools from three independent donors, each with varying frequencies of epitope-specific T cells, were co-cultured with epitope-loaded (specific) or nonloaded (nonspecific) target cells, and the fraction of apoptotic target cells was monitored. In all donors, epitope-specific cytotoxicity was observed across all conditions from 8% to 0.2% specific T cells, as indicated by a significant increase in apoptotic target cells in the epitope-loaded compared to the nonloaded condition. The three independent donors exhibited a comparable pattern of epitope-specific cytotoxicity, with a clear correlation between increasing frequencies of epitope-specific T cells and an earlier onset of target cell apoptosis (**Figure 4A**). Based on this indication that extended observation times may enhance the sensitivity of the assay, a prolonged monitoring period of 48 h enabled the detection of T-cell cytotoxicity mediated by as few as 0.1% specific T cells in donors 1 and 2 (**Figure 4B**). Reanalysis of the same experiments—focusing solely on changes in target cell area while disregarding caspase 3/7 labeling—produced similar, albeit less significant, results. Overall, this analysis strategy failed to detect cytotoxicity mediated by low frequencies of epitope-specific T cells, particularly for donor 2 (**Supplementary Figure 6**). These findings indicate that while an analysis based solely on changes in the target cell area is feasible, it lacks the sensitivity achieved with the dual staining approach using a caspase dye.

Supernatants from the cytotoxicity assays were collected and analyzed for common CD8^+^ T-cell cytokines to validate epitope-specific T-cell activation. After 48 h of T cell and target cell co-culture, IFN-γ levels were significantly increased in conditions containing 0.5% or more epitope-specific T cells and epitope-loaded target cells (**Figure 4C**). A slight increase in IFN-γ levels was also observed in the condition with only 0.2% epitope-specific T cells co-cultured with specific target cells. However, this increase was much less pronounced compared to the cytotoxicity observed in this condition, underscoring the high sensitivity of the cytotoxicity assay. Importantly, the IFN-γ levels validate the epitope-specific activation of T cells, as they correlate very well with the frequency of epitope-specific CD8^+^ T cells, while IFN-γ levels in unloaded control conditions remain at a consistently low level. Additionally, the levels of granzyme A and B, Fas, Fas-ligand, TNF-α, IL-4, IL10, and IL-6 in peptide-loaded conditions increased with the rising numbers of epitope-specific T cells, whereas the levels of perforin, granulysin, and IL-2 remained unaffected (**Supplementary Figure 7**). However, strong donor-dependent differences in basal cytokine release were observed for most of these cytokines.

Finally, CD8^+^ T cells of the 8% epitope-specific T-cell conditions were analyzed by flow cytometry for surface expression of PD-1 and LAG3 after the cytotoxicity assay. The gating strategy is shown in **Supplementary Figure 3**. Epitope-specific and nonspecific CD8^+^ T cells were differentiated by dextramer staining. Epitope-specific T cells showed a strong increase in the expression of PD-1 and LAG3 upon co-culture with epitope-loaded target cells, but not upon co-culture with unloaded target cells (**Figures 4D–I**). As expected, nonspecific T cells showed no increase in these markers, irrespective of target cell loading (**Supplementary Figure 8**). Especially surface expression of the activation/exhaustion marker LAG3 was almost nondetectable for nonspecific CD8^+^ T cells as well as for epitope-specific T cells that did not recognize their target epitope. A very specific upregulation of LAG3 was only detected upon activation of the CTLs by their specific epitope (**Figures 4G–I**). As PD-1 is already expressed in T cells, and expression increases during specific target cell contact (**Figure 4D**), this difference can be more clearly seen in the mean fluorescence intensity (MFI) analysis (**Figure 4E**). LAG3 expression, on the other hand, is induced by specific target cell contact (**Figure 4G**), thus the effect is more clearly visible in the frequency analysis (**Figure 4I**).

To assess whether the assay is sufficiently sensitive to detect epitope-specific cytotoxicity directly *ex vivo*, we used PBMCs from donors with known CMV reactivity. Indeed, significant target cell killing by as few as 0.18% epitope-specific T cells was detected (**Supplementary Figure 5**). As expected, the effect size was smaller than that observed with expanded T cells; however, due to the very low background in the *ex vivo* setting, specific killing was still clearly observed.

In summary, the data validate the high sensitivity of cytotoxicity detection achieved with this assay setup and confirm the epitope-specific activation of CTLs in the assay co-culture.

### 3.4 Detection of epitope-specific T-cell cytotoxicity mediated by rare HPV16 epitopes

**Figure 5:**
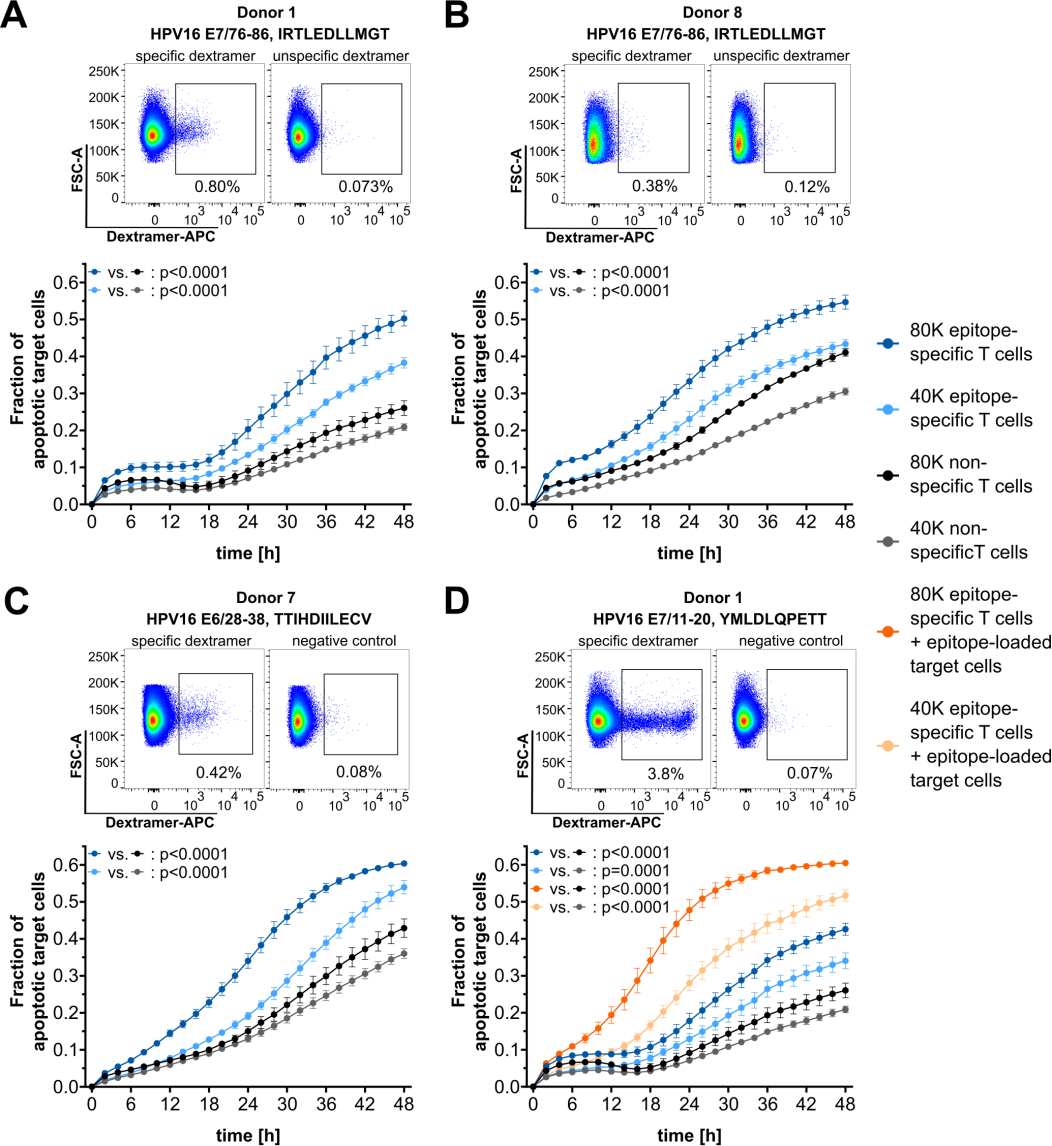
Functional validation of HPV16-derived epitopes. **(A–D)** Top: Dextramer stainings for epitope-specific CD8^+^ T cells (left) and corresponding negative control staining with unspecific dextramers (right). Bottom: Corresponding cytotoxicity assay results. Target cell line: SNU1299. Epitopes and T-cell numbers are indicated. The mean of four technical replicates for each timepoint is shown, with error bars indicating the SD. Repeated measures ANOVA was used for significance testing. *p*-values are shown.

The high sensitivity of this cytotoxicity assay enables the functional validation of rare CTL populations. One possible application of this assay is the validation of human papillomavirus (HPV)16 E6- or E7-derived epitopes, against which T-cell populations are extremely rare in the peripheral blood of healthy donors (20, 21). T cells from buffy coat preparations with known reactivity to A*02:01-restricted HPV16-derived epitopes were *in vitro* expanded using the respective peptides, and T-cell frequencies were determined by dextramer staining (**Figure 5**, top of all panels). Background control-corrected epitope-specific T-cell frequencies based on dextramer stainings ranged from 0.26% to 3.73%, which falls within the range of required T-cell frequencies for the cytotoxicity assay, as determined above. However, it should be noted that the quality of dextramer staining also depends on the loading efficiency, which is very low for E7/76-86 (**Supplementary Figure 1**). Despite the very low frequencies of epitope-specific T cells detected by dextramer stainings, highly significant epitope-specific cytotoxicity against naturally HPV16-transformed target cells was observed for E7/76–86 using T cells from two independent donors (**Figures 5A, B**). Given the observed increase in assay sensitivity with extended monitoring times when working with low frequencies of specific T cells (c.f. **Figure 4B**), a 48-h monitoring period was used in the HPV assays. Additionally, epitope-specific cytotoxicity was detected for the peptides E6/28-38 (**Figure 5C**) and E7/11-20 (**Figure 5D**). For E7/11-20, which strongly binds to A*02:01, an additional condition with artificially epitope-loaded target cells resulted in increased cytotoxicity. This underscores that enhanced presentation of the target epitope on tumor cells can improve recognition by CTLs.

## 4 Discussion

Overall, the term cytotoxicity assay refers to two different types of *in vitro* analyses: toxicity assays, which are used to assess cell death induced by specific treatments (e.g., compound screenings), and assays that focus on the cell-mediated killing of a second cell population. These two types of cytotoxicity assays involve different requirements.

Non-cell-mediated cytotoxicity can be assessed by several different assays. These are usually based on the loss of membrane integrity, which allows selective penetration of dead cells by vital dyes or the release of exclusively intracellular proteins. Probably the most common approach to identify dead cells is trypan blue staining, but several fluorescent DNA-binding dyes are also used and are commercially available. Similarly, a variety of kits are available to quantify the release of intracellular proteins, thereby measuring the cytotoxicity of a given treatment (5).

While these assay strategies are straightforward approaches for analyzing the cytotoxicity of noncellular treatments, the assay setup and readout become significantly more complex when a second cell population is introduced to assess cell-mediated cytotoxicity. Once a co-culture system with effector and target cells is used, the assay must be meticulously controlled, as dying effector cells can also contribute to the observed signal. Consequently, proper controls are needed, which typically doubles the required number of effector cells. This can be addressed by introducing an artificial label to the target cells only, as was done in the traditional ^51^Cr release assay (1). However, since this method involves the use of radioactive material, it is increasingly being replaced. For example, several FACS-based cytotoxicity assay strategies have been developed that enable the discrimination between target and effector cells, making them well-suited for assessing cell-mediated cytotoxicity (6, 22).

For both types of cytotoxicity assay strategies—noncellular and cell-mediated—the development of new methods, such as impedance measurements and live-cell imaging techniques, has enabled the monitoring of cell death over time. However, in these assay approaches, the assessment of cell-mediated cytotoxicity still requires a reliable distinction between effector and target cells. This requirement is inherently addressed in impedance-based assays, where only the detachment of adherent target cells—unlike the presence of nonadherent effector cells—affects the results obtained (9). In contrast, imaging-based assays require additional labeling of the target cells. Furthermore, imaging-based assay setups provide the opportunity not only to monitor the area covered by target cells but also to combine this with additional stainings—e.g., to detect cells that have lost membrane integrity or to specifically stain for apoptotic cells using caspase markers.

Here, we made use of the advantages of imaging-based assays and established a highly sensitive live-cell imaging-based cytotoxicity assay setup, optimized for the analysis of epitope-specific cell-mediated cytotoxicity executed by low effector cell frequencies. The use of transient labeling of the target cell population enables reliable differentiation between target and effector cells, while also allowing a fast and easy adaptation of the assay to different target cell populations. Additionally, the use of a caspase-3/7 labeling dye allows more sensitive detection of cells undergoing apoptosis, thereby increasing assay sensitivity compared to analyses based solely on changes in the target cell area. Our dual staining-based assay setup demonstrated the ability to detect epitope-specific cytotoxicity mediated by as few as 0.1% of CTLs within a T-cell pool. A comparable level of sensitivity has previously been reported for the flow cytometry-based cytotoxicity assay “Vital-FR”, which also targets the detection of epitope-specific cytotoxicity (6). Nevertheless, there are pronounced differences between the assay presented here and the Vital-FR assay, making each method suitable for different applications. A primary distinction is that the Vital-FR assay was developed for suspension target cells, whereas our assay was designed for the analysis of adherent target cells. We chose to focus on adherent target cells to facilitate the analysis of cytotoxicity against endogenously presented peptides on tumor cell lines derived from solid tumors. This approach limits the possible target cells to those that adhere well within 24 h of seeding. However, since the use of suspension cells is feasible in live-cell imaging-based assays (23), it may be possible to adapt our assay for nonadherent target cells as well.

The most important difference between our setup and the Vital-FR assay is that the latter requires the mixing of two closely matched target cell lines. This can be achieved by loading identical target cells with either the test epitope or an unrelated control epitope, as described by Stanke et al., or by using genetically engineered cell lines of the same origin that differ only in the expression of the relevant antigen(s) [as applied in (24)]. However, both of these options are not applicable to the analysis of cytotoxicity against unmodified patient-derived (tumor) cell lines. In such cases, the best strategy is to use closely HLA-matched cell lines—by necessarily derived from different donors—with and without the target antigen [as applied in (25)], though finding such combinations is challenging.

Therefore, we decided to design an assay setup that does not rely on a co-culture system involving two different target cell lines, in order to allow the analysis of endogenously presented epitopes on naturally transformed tumor cell lines. In addition, the motivation for this study was to develop a method for assessing the cytotoxicity of rare T-cell populations without requiring prior enrichment steps, such as FACS- or MACS-based sorting. This approach also eliminates the need to generate multimers for every candidate peptide to be analyzed, thereby facilitating larger-scale screenings for promising target epitopes. However, using T-cell populations that contain only low fractions of epitope-specific T cells presents certain challenges. On the one hand, the epitope-specific effects are weak; on the other hand, background effects caused by the high number of T cells can be substantial. These background effects depend on the activation state of the CD8^+^ T cells used, as well as on the respective T-cell donor. While e*x vivo* analysis of CMV-specific cytotoxicity showed almost no background effects (but also only weak epitope-specific cytotoxicity), experiments with *in vitro* expanded and activated T cells demonstrated significant epitope-specific killing but stronger background effects and high donor-dependent variability. Donor-dependent variability may result from several factors, such as differing responses to *in vitro* expansion protocols (if used), variations in the effector states of the CD8^+^ T cells, and differing levels of alloreactivity against the target cells due to HLA mismatch. These factors can influence the assay’s outcome and may limit its applicability if donor-dependent background effects are too pronounced to detect epitope-specific responses.

To account for the low frequency of epitope-specific T cells, T cell-to-target cell ratios of 4:1 and 2:1 were used in this study. Compared to many other cytotoxicity assay applications, these ratios are relatively high, indicating that a large number of T cells is used. However, since the assay setup targets T-cell populations with low frequencies of epitope-specific cells, these T cell-to-target cell ratios are not equivalent to common effector-to-target (E:T) ratios, as the majority of the T cells are not epitope-specific. Titration of epitope-specific T cells demonstrated that epitope-specific killing mediated by only 0.1% specific CD8^+^ T cells can still be detected at a T cell-to-target cell ratio of 2:1, which corresponds to an epitope-specific T cell-to-target cell ratio of 0.002:1. In experiments using a T-cell population with 97% antigen specificity, similar or lower sensitivity has been reported for impedance-based assays and CRA, with E:T ratios of 0.005:1 and 0.05:1, respectively (9).

Due to the observed background effects in the co-culture of *in vitro* activated T cells and only partially HLA-matched target cell lines (see **Supplementary Table 3**), it is necessary to control for these background effects on the T-cell side. We therefore expanded donor PBMCs either against the peptide of interest or against a control peptide that is not naturally presented by the target cells used. This approach generated two cell pools with the same background but differing in their fractions of epitope-specific T cells. The cytotoxicity elicited by these two T-cell pools was then compared to identify truly epitope-specific cytotoxicity. To validate this strategy, we selected HPV epitopes as a representative model, characterized by low frequencies of epitope-specific T cells in peripheral blood and low expression and presentation of these epitopes in naturally transformed tumor cell lines. We successfully detected epitope-specific cytotoxicity of healthy donor PBMCs against three endogenously presented HPV16 epitopes. The E7/11–20 epitope is a well-known HPV16-derived epitope with a strong binding affinity to HLA-A*02:01, for which immunogenicity and epitope-specific CTL cytotoxicity were described long ago (26, 27). Our data are consistent with these earlier findings, thereby validating both our results and the assay setup. Additionally, artificial pulsing of the target cells with this epitope increased the observed cytotoxicity, as expected effect with enhanced epitope presentation. The E7/76–86 epitope has also previously been shown to be immunogenic in healthy donors (28). In our study, we observed cytotoxicity of E7/76-86-specific CTLs in two healthy donors, thereby demonstrating that this peptide can successfully mediate target cell killing. Furthermore, we demonstrated E6/28-38-specific cytotoxicity in one donor, representing the first functional validation of this HPV16-derived epitope. Moreover, the observed cytotoxicity indicates that the cervical cancer cell line SNU1299 naturally presents the epitopes E7/11-20, E7/76-86, and E6/28-38.

In conclusion, we demonstrated that this highly sensitive live-cell imaging-based cytotoxicity assay enables time-course analysis of epitope-specific T-cell killing. It is suitable for detecting epitope-specific cytotoxicity mediated by rare CD8^+^ T-cell populations, even in the presence of other CD8^+^ T cells with different specificities. This approach allows for the analysis of epitope-specific cytotoxicity by low-abundance T-cell populations directly *ex vivo* from blood or after simple *in vitro* expansion, without requiring additional enrichment steps. Together with the ability to assess T-cell responses against endogenously presented epitopes offers a valuable advantage for analyzing cancer- or virus-specific T-cell cytotoxicity.

## Data Availability

The original contributions presented in the study are included in the article/supplementary material. Further inquiries can be directed to the corresponding author.
