# Supplementary material for: Highly sensitive live-cell imaging-based cytotoxicity assay enables functional validation of rare epitope-specific CTLs
Source: Front Immunol. 2025 May 8;16:1558620. doi: 10.3389/fimmu.2025.1558620 (PMC12095279; doi:10.3389/fimmu.2025.1558620)
Supplement: Supplementary file 1 [file DataSheet1.pdf]

Highly sensitive live-cell imaging-based cytotoxicity assay enables functional validation of rare epitope-specific CTLs

Suppl. Table 1: Peptide sequences and origin

ID	Sequence	Virus	Source protein	Amino acid position	Ligand to
EBV-A2	GLCTLVAML	EBV	BMLF1	280-288	HLA-A02*01
CMV-A2	NLVPMVATV	CMV	pp65	495-503	HLA-A02*01
CMV-A11	ATVQGQNLK	CMV	pp65	501-509	HLA-A11*01
CMV-A1	YSEHPTFTSQY	CMV	pp65	363-373	HLA-A01*01
E7/76-86	IRTLEDLLMGT	HPV16	E7	76-86	HLA-A02*01
E6/28-38	TTIHDIILECV	HPV16	E6	28-38	HLA-A02*01
E7/11-20	YMLDLQPETT	HPV16	E7	11-20	HLA-A02*01

Suppl. Table 2: Cell culture media

	Ingredients	Article number	Supplier	Concentration [%]
**CaSki medium**	DMEM	D5796	Sigma Aldrich	88
	Fetal bovine serum (FBS)	A5256801	Sigma Aldrich	10
	Penicillin/Streptomycin (10,000 units/mL / 10 mg/mL)	P0781	Sigma Aldrich	1
	L-Glutamine (200 mM)	25030123	Thermo Fisher Scientific	1
**Cytotox assay medium/ SNU902 + SNU1299 medium**	RPMI	31870074	Thermo Fisher Scientific	87
	FBS	A5256801	Sigma Aldrich	10
	Penicillin/Streptomycin (10,000 units/mL / 10 mg/mL)	P0781	Sigma Aldrich	1
	L-Glutamine (200 mM)	25030123	Thermo Fisher Scientific	1
	HEPES (1M)	15630056	Thermo Fisher Scientific	1
**T cell medium**	RPMI 1640	31870074	Thermo Fisher Scientific	87
	Human serum	H4522	Sigma Aldrich	10
	Gentamicin (10 mg/mL)	15710049	Thermo Fisher Scientific	1
	L-Glutamine (200 mM)	25030123	Thermo Fisher Scientific	1
	HEPES (1M)	15630056	Thermo Fisher Scientific	1

Suppl. Table 3: HLA class I profile of target cell lines and donor PBMCs

**Cells**	**HLA-A_I**	**HLA-A_II**	**HLA-B_I**	**HLA-B_II**	**HLA-C_I**	**HLA-C_I**
SNU1299	02:01	33:03	27:05	44:03	01:02	14:03
SNU902	01:01	11:01	07:05	35:01	03:EEDAC*	15:05
CaSki	02:01	03:01	07:02	37:01	07:EEDAV*	07:EEDAV*
Donor 1	02:01	32:01	08:01	44:02	05:01	07:01
Donor 2	02:01	32:01	07:02	27:05	02:CEJWX*	07:CENAH*
Donor 3	11:01	23:01	44:02	44:03	04:01	07:04
Donor 4	01:01	03:01	07:02	51:01	07:DXFTV*	14:02
Donor 5	01:01	02:01	08:01	18:01	07:01	07:01
Donor 6	01:01	02:01	14:01	51:01	08:02	15:ECXKC*
Donor 7	02:01	11:01	40:01	44:03	03:CEJXD*	16:01
Donor 8	02:01	30:01	07:02	44:03	07:02	16:01
Donor 9	01:01	02:01	40:01	40:01	03:CEJXD	03:CEJXD
Donor 10	02:01	03:01	13:02	44:02	05:01	06:02

*HLA-typing could not identify specific alleles, possible alleles are given as MAC codes

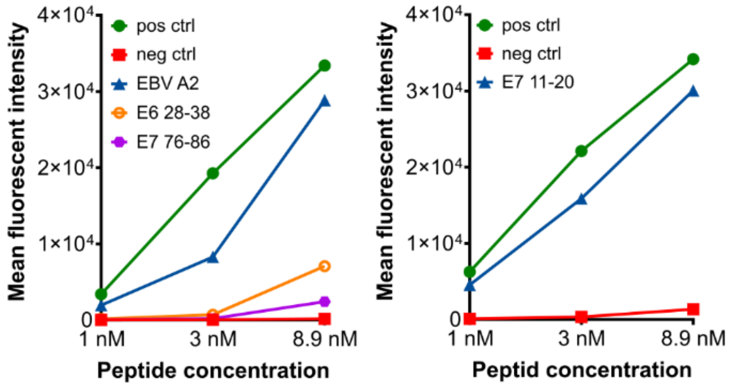

Suppl. Figure 1: Loading efficiency analysis of u-load easYmers. The loading efficiency of all used easYmers was assessed in comparison to a positive (strong binder) and a negative (non-binder) control peptide.

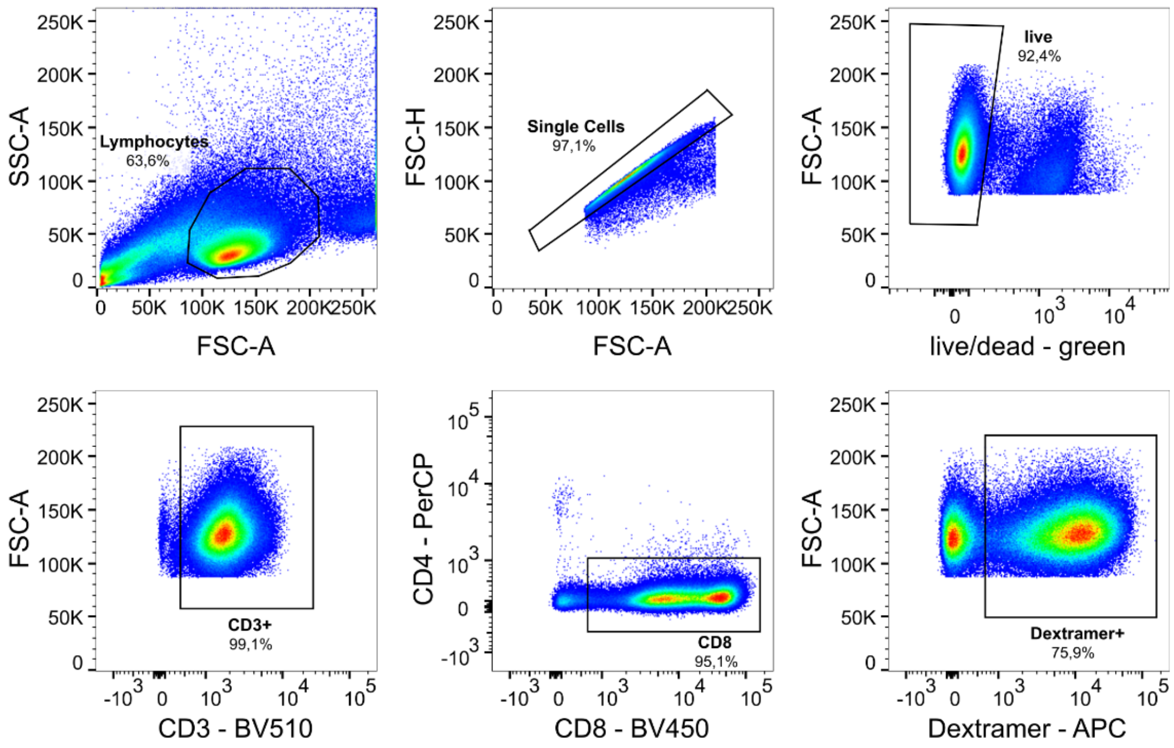

Suppl. Figure 2: Gating strategy for epitope-specific CTLs. First, lymphocytes were selected based on their properties in the SSC-A – FSC-A plot. This population was gated for single cells by excluding doublets with decreased FSC-H values and subsequently live cells with low live/dead-green signal were selected. From all live cells, $CD3^+$ cells were identified based on a strong BV510 signal compared to unstained control. Similar, $CD8^+$ within the population of $CD3^+$ cells were gated based on the BV450 signal. As expected, the frequencies of $CD3^+$ and $CD8^+$ cells were very high, as the staining was performed after $CD8^+$ T cell enrichment by MACS. Finally, the frequency of epitope-specific CTLs was assessed by analyzing the frequency of dextramer-APC positive $CD8^+$ T cells compared to a negative dextramer control (exemplarily shown for EBV-A2$^+$ $CD8^+$ T cells of donor 1).

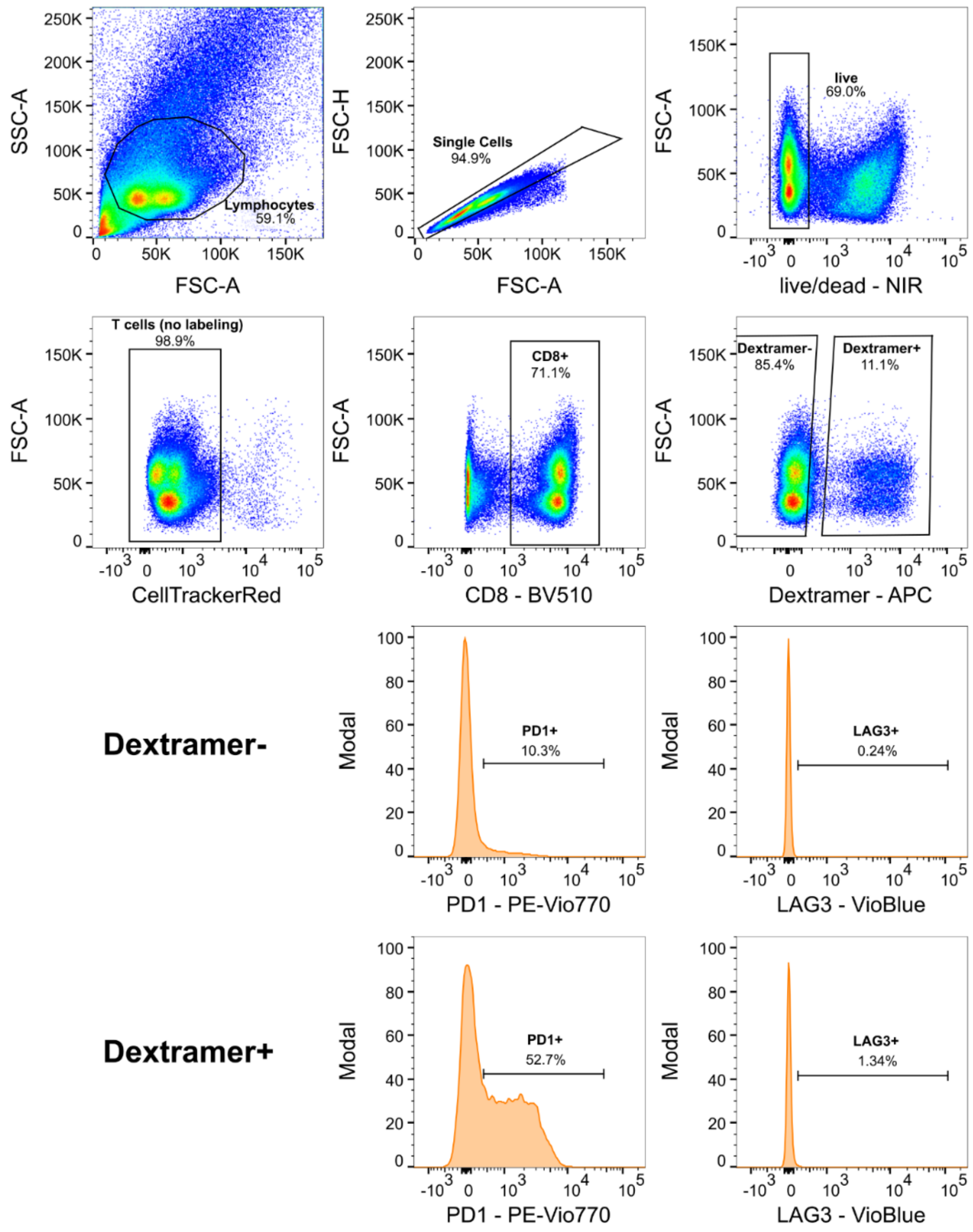

Suppl. Figure 3: Gating strategy for PD1 and LAG3 expression of epitope-specific and non-specific CTLs. First, lymphocytes were selected based on their properties in the SSC-A – FSC-A plot. This population was gated for single cells by excluding doublets with decreased FSC-H values and subsequently live cells with low live/dead-green signal were selected. To ensure that no target cells were accidentally included in the live cell population, cells with a CellTrackerRed signal were excluded. Then, $CD8^+$ cells were gated based on the BV510 signal. Finally, gates for epitope-specific ($dextramer^+$) and non-specific ($dextramer^-$) populations were set. The same gates for PD1-PE-Vio770 and the LAG3-VioBlue were applied to both populations (exemplarily shown for $CD8^+$ T cells of donor 1 after co-culture with unspecific target cells).

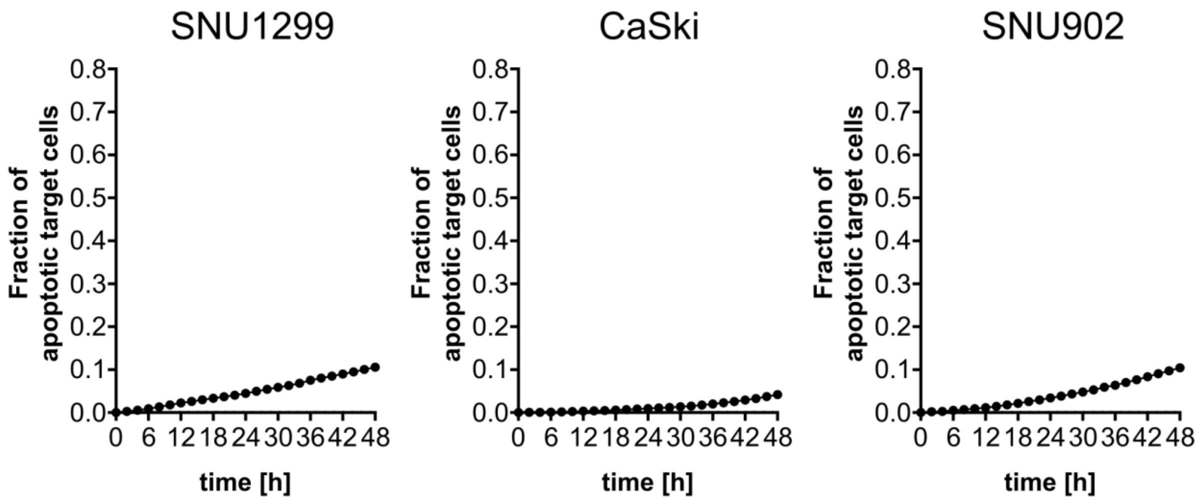

Suppl. Figure 4: Analysis of the fraction of apoptotic target cells transiently labeled with CellTrackerRed. Target cell lines were transiently labeled, seeded in the same concentrations as used for the cytotoxicity assays and cultured in medium containing caspase 3/7 dye. Mean values of n=9 technical replicates are shown. The standard deviation is to small to be shown as error bars.

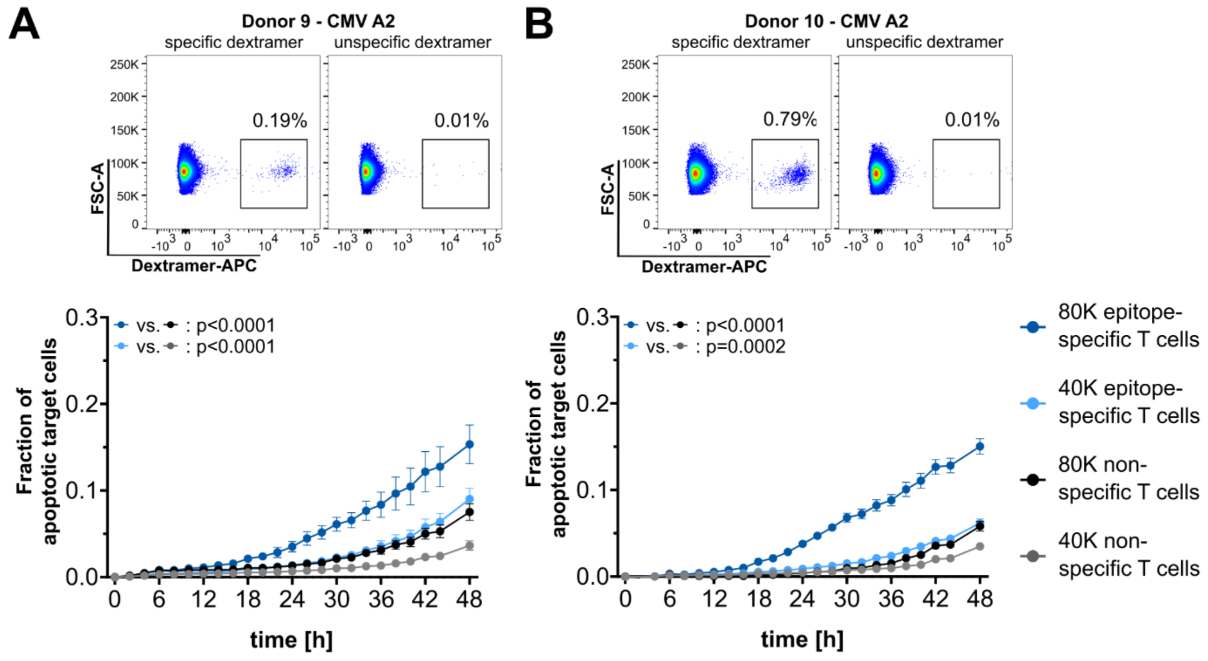

Suppl. Figure 5: *Ex vivo* cytotoxicity analysis of CMV-A2-specific $CD8^+$ T cells. A+B: Top: Dextramer stainings for CMV-A2-specific $CD8^+$ T cells (left) and corresponding negative control staining with unspecific dextramers (right). Bottom: Corresponding cytotoxicity assay results. Model epitope: CMV-A2 (NLVPMVATV**)**. Target cell line: CaSki. T cell number per well: 40,000. The mean of four technical replicates for each timepoint is shown with error bars indicating the SD. Repeated measures ANOVA was used for significance testing. p-values are shown.

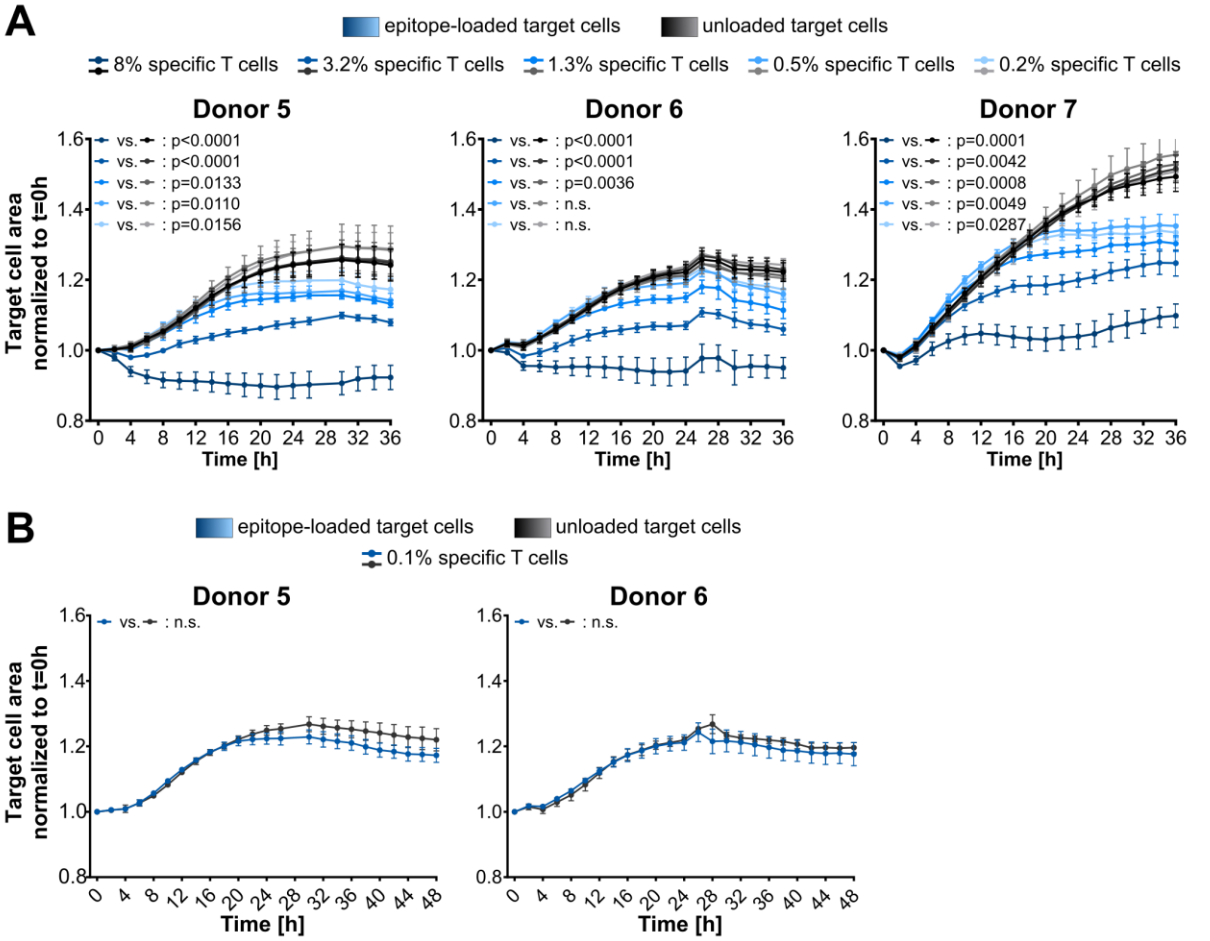

Suppl. Figure 6: Assay sensitivity analysis based on the red target cell area. A+B: Cytotoxicity assay plots with titration of the frequency of epitope-specific $CD8^+$ T cells in three (A) or two (B) independent HLA-A*02:01 expressing donors. Model epitope: EBV-A2 (GLCTLVAML). Target cell line: CaSki. T cell number per well: 40,000. EBV-A2-specific T cell frequencies were identified by dextramer staining and desired frequencies were set by combination with non-epitope-specific $CD8^+$ T cells from the respective donor. The mean of four technical replicates for each timepoint is shown with error bars indicating the SD. Repeated measures ANOVA was used for significance testing. p-values are shown. n.s. indicates non-significant results.

Note: As this analysis is only based on the target cell area (normalized to t=0h by division), enhanced killing, which leads to a decrease of the target cell area, results in lower curves.

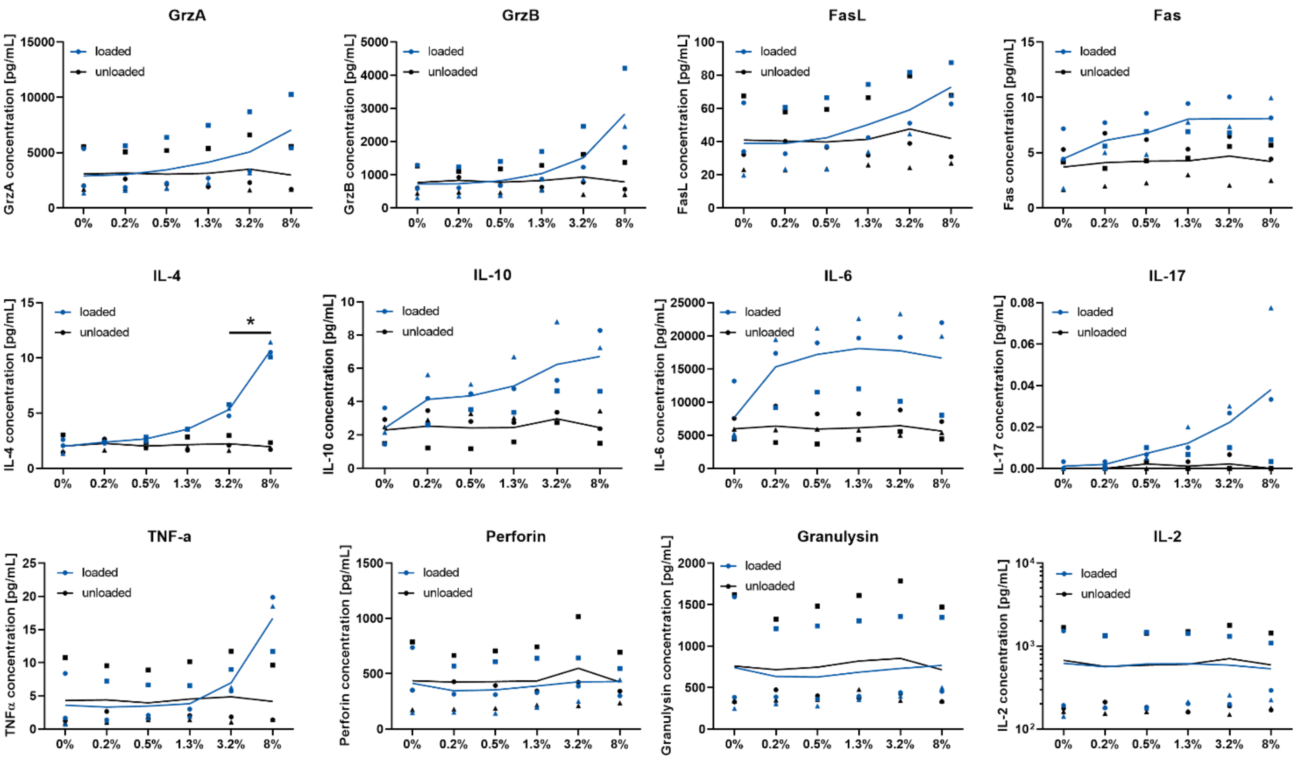

Suppl. Figure 7: Cytokine release profile after cytotoxicity assay titration experiments. Cytokine concentrations in cytotoxicity assay supernatant after 48h of T cell – target cell co-culture with various frequencies of epitope-specific T cells (indicated on x-axis). Different donors are indicated with different symbols: ●: Donor 5, ▲: Donor 6, ■: Donor 7. Multiple t-tests with Holm-Sidak correction for multiple comparisons were used tor significance testing. Significant differences with p-values < 0.05 are highlighted with an asterisk.

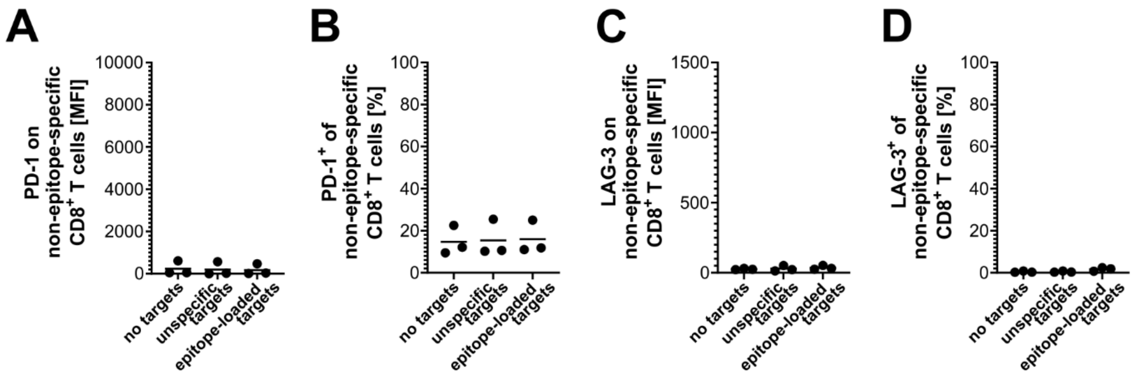

Suppl. Figure 8: PD-1 and LAG-3 surface expression on non-epitope-specific CD8$^+$ T cells. A: Mean fluorescence intensity (MFI) of PD-1-PEVio770. B: Frequency of PD-1-PEVio770 positive CD8$^+$ T cells. C: Mean fluorescence intensity (MFI) of LAG3-VioBlue. D: Frequency of LAG3-VioBlue positive CD8$^+$ T cells.
